# Adulterations of Food

**Published:** 1874-11

**Authors:** 


### ADULTERATIONS OF FOOD.

WE shall commence in our January number a series of carefully prepared articles on the adulterations of food. These articles will be finely illustrated, and will give the most simple and effective means for the detection of adulterations in articles of daily use. They will be widely read, and cannot fail to do great good.

